# Why We Eat What We Eat: Assessing Dispositional and In-the-Moment Eating Motives by Using Ecological Momentary Assessment

**DOI:** 10.2196/13191

**Published:** 2020-01-07

**Authors:** Deborah Ronja Wahl, Karoline Villinger, Michael Blumenschein, Laura Maria König, Katrin Ziesemer, Gudrun Sproesser, Harald Thomas Schupp, Britta Renner

**Affiliations:** 1 Psychological Assessment and Health Psychology Department of Psychology University of Konstanz Konstanz Germany; 2 Data Analysis and Visualization Department of Computer and Information Science University of Konstanz Konstanz Germany; 3 General and Biological Psychology Department of Psychology University of Konstanz Konstanz Germany

**Keywords:** mHealth, eating, motivation, mobile app, EMA, in-the-moment, disposition, trait, state

## Abstract

**Background:**

Why do we eat? Our motives for eating are diverse, ranging from hunger and liking to social norms and affect regulation. Although eating motives can vary from eating event to eating event, which implies substantial moment-to-moment differences, current ways of measuring eating motives rely on single timepoint questionnaires that assess eating motives as situation-stable dispositions (traits). However, mobile technologies including smartphones allow eating events and motives to be captured in real time and real life, thus capturing experienced eating motives in-the-moment (states).

**Objective:**

This study aimed to examine differences between why people think they eat (trait motives) and why they eat in the moment of consumption (state motives) by comparing a dispositional (trait) and an in-the-moment (state) assessment of eating motives.

**Methods:**

A total of 15 basic eating motives included in The Eating Motivation Survey (ie, liking, habit, need and hunger, health, convenience, pleasure, traditional eating, natural concerns, sociability, price, visual appeal, weight control, affect regulation, social norms, and social image) were assessed in 35 participants using 2 methodological approaches: (1) a single timepoint dispositional assessment and (2) a smartphone-based ecological momentary assessment (EMA) across 8 days (N=888 meals) capturing eating motives in the moment of eating. Similarities between dispositional and in-the-moment eating motive profiles were assessed according to 4 different indices of profile similarity, that is, overall fit, shape, scatter, and elevation. Moreover, a visualized person × motive data matrix was created to visualize and analyze between- and within-person differences in trait and state eating motives.

**Results:**

Similarity analyses yielded a good overall fit between the trait and state eating motive profiles across participants, indicated by a double-entry intraclass correlation of 0.52 (*P*<.001). However, although trait and state motives revealed a comparable rank order (*r*=0.65; *P*<.001), trait motives overestimated 12 of 15 state motives (*P*<.001; *d*=1.97). Specifically, the participants assumed that 6 motives (need and hunger, price, habit, sociability, traditional eating, and natural concerns) are more essential for eating than they actually were in the moment (*d*>0.8). Furthermore, the visualized person × motive data matrix revealed substantial interindividual differences in intraindividual motive profiles.

**Conclusions:**

For a comprehensive understanding of why we eat what we eat, dispositional assessments need to be extended by in-the-moment assessments of eating motives. Smartphone-based EMAs reveal considerable intra- and interindividual differences in eating motives, which are not captured by single timepoint dispositional assessments. Targeting these differences between why people think they eat what they eat and why they actually eat in the moment may hold great promise for tailored mobile health interventions facilitating behavior changes.

## Introduction

### Background

Food is almost ubiquitous in our everyday life, and *eating* is one of the simplest yet most complex behaviors [1-3], involving up to 200 decisions a day [4]. The motives for and functions of eating in everyday life play a crucial role in promoting healthy eating behaviors [5]. A deeper understanding of the underlying mechanisms and causes of human food choices is indispensable for designing and facilitating effective primary interventions to counteract the obesity epidemic [6] and its associated health risks [7-9]. The questions “what we eat” and also “why we eat, what we eat” are therefore of great importance for promoting normal eating behavior and preventing the development of obesity and eating disorders.

Everyday human eating behaviors are regulated by numerous motives [10,11] that range from physiological factors [12,13], psychological factors (such as positive or negative emotional states [14-17] and social reasons [18-20], to various situational factors such as food’s smell or appearance [21-24]. Thus, in addition to hunger, there are other compelling reasons for choosing and eating certain food items.

As eating motives are multidimensional, assessing eating motives is a major challenge. Most psychometric measures focus on specific motives, such as the Motivation to Eat Scale, which assesses 4 core motives (pleasure, coping with negative affect, being social, and complying with others’ expectations [25]), or the Dutch Eating Behavior Questionnaire, which includes eating in response to negative emotions and in response to external sensory cues as 2 core motivations for eating [24]. A more comprehensive conceptualization of eating motives is provided by the Food Choice Questionnaire (FCQ), which encompasses 9 different food choice motives for everyday life of which the taste, appearance, and smell of food were rated as the most important motives for food choices, followed closely by healthiness, affordability, and availability [26-29]. However, as the FCQ does not include important motives such as social or physiological motives, The Eating Motivation Survey (TEMS) was developed to cover a more extensive set of 15 basic eating motives [10,11], including eating because of liking, habit, need and hunger, health concerns, convenience, pleasure, tradition, natural concerns, price considerations, visual appeal, sociability, weight control concerns, negative affect regulation, and concerns about social norms and social image, which have been found consistently across different groups [10,30], contexts [31], and countries [11,32].

Although these current psychometric measures capture multiple motives, they commonly assess eating motives as time and situational invariant dispositions (or traits), asking for *typical* reasons, for example, why respondents usually eat what they eat [10,29]. These dispositional measures capture why people think they eat what they eat. However, daily eating situations can differ greatly, for example, depending on time and place [15,33,34]. It is, therefore, likely that eating motives will vary in the moment of eating, both across and within individuals.

The rise of mobile health (mHealth) and mobile technology in medicine and public health offers great possibilities for capturing in-the-moment experiences, including eating motives in both real life and real time [22,33-38]. Smartphones are a particularly promising method of assessing eating events and eating motives in the moment because of the high level of global penetration and the ease of installing apps in all kinds of mobile devices [39,40]. Assessing eating motives in the moment offers important conceptual and methodological advantages compared with classical single timepoint dispositional measures [41]. Specifically, participants do not need to recall eating motives for each past eating event to derive a judgment about their typical eating motives. Considering the daily multitude of eating occasions, people are unlikely to accurately recall all their relevant reasons for eating and so must reconstruct their eating motives when gauging their typical eating motives [42-47]. Even when people manage to do this accurately, they still need to aggregate the different reasons across multiple eating occasions to infer their typical reasons [48]. Initial evidence on self-to-peer comparisons of eating motives [49] shows that people might have biased conceptions of their dispositional eating motives. Hence, current dispositional measures for assessing eating motives may be substantially affected by memory and aggregation biases [48,50].

Using an in-the-moment, that is, event-based, ecological momentary assessment (EMA) approach [38,51-53], eating motives can be repeatedly and comprehensively assessed when an eating event occurs, which ensures a high measurement accuracy and maximizes ecological validity [50,54-56]. In contrast to signal-content protocols, participants in event-based protocols determine for themselves when the event occurs and initiate an assessment [57,58]. Some approaches are even developing methods to automatically detect an eating event, see for example eButton [59] or automatic ingestion monitor [60]. This alleviates the problem of memory and aggregation bias associated with the conventional “single-shot” assessment of eating motives. However, research determining the correspondence of in-the-moment assessments with single timepoint dispositional measures is scarce [51,61-64] and to our knowledge has not been addressed with regard to eating events and their underlying motives.

### This Study

The aim of this study was to examine differences between why people think they eat (trait motives) and why they eat in the moment of consumption (state motives) by assessing 15 different basic eating motives [10] measured by (1) a single timepoint dispositional (trait) and (2) a 1-week in-the-moment (state) assessment of eating motives using smartphone-based EMA [52,54,65].

Profile similarity indices are used to analyze whether and to what degree the 15 trait eating motives concur with the 15 state motives [66,67]. The omnibus index reflects a proxy of the overall fit between the trait and state eating motive profiles. Furthermore, profiles can be similar in respect of 3 major characteristics: their shape, scatter, and elevation. A large shape similarity indicates that the same motives score on average high (or low) within the trait and state profile. A large scatter similarity indicates that the variability between the 2 eating motive profiles is relatively comparable. A high elevation similarity indicates that the average of the 15 motives is similar between the trait and state measure [66,67]. These similarity indices can be applied on different levels of analyses to assess inter- and intraindividual differences in profile similarity, including the between-person, between-motive, and the within-person level.

We developed a new visualization tool called the *SMART-Profile-Explorer* to comprehensively analyze and visualize high-dimensional data. The Web-based SMART-Profile-Explorer can be used interactively to sort, filter, and visualize these data, making the data available to other scientists and facilitating communication and data sharing [68].

## Methods

### Study Guidelines

This study was part of the research project, SMARTACT, funded by the German Federal Ministry of Education and Research (Bundesministerium für Bildung und Forschung). The study adhered to the guidelines of the German Psychological Society (Deutsche Gesellschaft für Psychologie) and the Declaration of Helsinki. The University of Konstanz’s Institutional Review Board approved the study protocol, and it is in accordance with ethical guidelines and regulations. All participants gave written informed consent before participation.

### Participants

In total, 35 individuals participated in the study (88.6% female, *n*=31), with a mean age of 25.49 years (SD 5.70; range 19-41 years) and an average body mass index of 22.51 kg/m^2^ (SD 5.51; range 15.43-42.87 kg/m^2^). No participants dropped out of the study.

### Procedure

**Figure 1:**
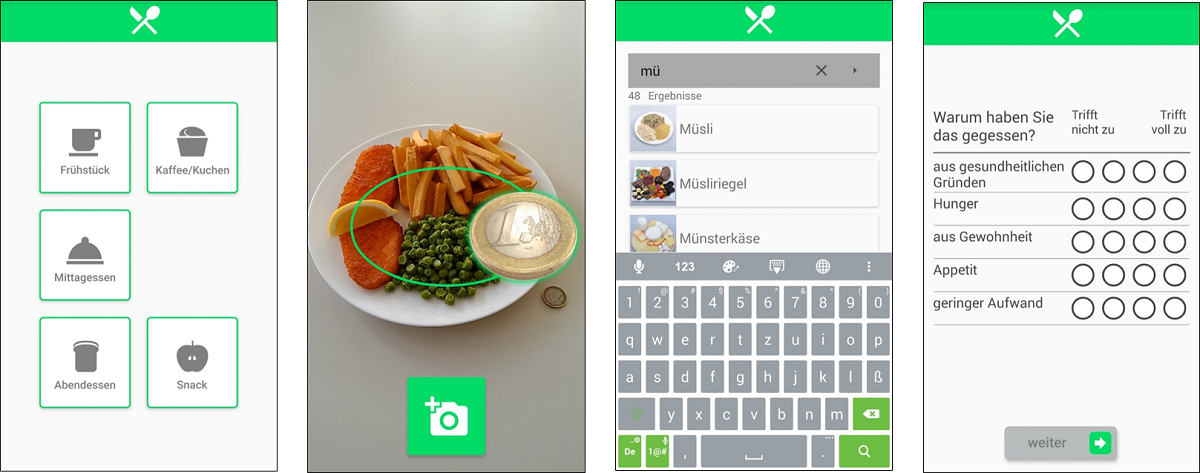
Screenshots of the SMARTFOOD app for assessing eating events and the 15 eating motives.

Participants were recruited through leaflets distributed at the University of Konstanz and postings on Facebook groups. Participants were invited to the laboratory for individual introductory sessions. At the baseline session, participants completed a questionnaire assessing 15 dispositional (trait) eating motives based on a single-item version of TEMS [10] and demographic variables. We used the mobile app, SMARTFOOD, which was developed as part of the research project SMARTACT [69], to record these motives in the moment of consumption [36]. The participants were provided and familiarized with the smartphones (ASUS Padphone Infinity, Android 5.0.2) and research app during the introductory session. They were asked to record all eating events for 8 consecutive days using the SMARTFOOD app (Figure 1). Specifically, they were asked to record the meal type (Figure 1, left), to take a picture of their meal (Figure 1, second from left), and to classify what they ate using a drop-down menu (Figure 1, third from left). Additional courses and leftovers were also recorded by taking pictures. In addition, participants rated 15 reasons why they ate what they ate based on the brief TEMS (Figure 1, right). As compensation, participants could choose between receiving €25 or course credits (3 hours).

### Measures

The 15 eating motives were surveyed using a single-item version of TEMS (see Multimedia Appendix 1), in which a single item represented each of the motives, including *liking, habit, need and hunger, health, convenience, pleasure, traditional eating, natural concerns, sociability, price, visual appeal, weight control, affect regulation, social norms,* and *social image* [10]. In the single timepoint dispositional assessment (trait motives) and at every time participants logged a meal or snack (state motives), they were asked to answer the question “I eat what I eat because of...” by rating each of the 15 motives on a Likert scale ranging from (1) “strongly disagree” to (4) “strongly agree.”

### Analytical Procedure

To statistically compare trait with state eating motives, repeatedly assessed state motives were averaged across all eating occasions. Profile similarity was analyzed according to 4 different similarity indices: overall profile similarity by double-entry intraclass correlations (*ICC_de_*), shape similarity (*r*) by Pearson correlations, scatter similarity (*Var_D_*) by raw differences between profile variances, and elevation similarity (*M_D_*) by raw differences between profile means [66,67]. Findings were analyzed by paired *t* tests and Pearson correlation analyses for each motive. Effect sizes were classified by using Cohen *d* [70]. All analyses were conducted with IBM SPSS (*version 24*). Furthermore, the SMART-Profile-Explorer was used to visualize and compare the resulting trait and state eating motive profiles at the different levels of data analyses [71] [72].

## Results

### Eating Occasions

In total, 888 eating occasions were recorded during the 8-day EMA period. By using a participant-identified approach [34,73], 231 (25.8%) eating occasions were classified as breakfast, 194 (21.8%) as lunch, 25 (2.8%) as afternoon tea, 209 (23.5%) as snacks, and 229 (25.8%) as dinner.

### State and Trait Eating Motives: The Visualized Person × Motive Data Matrix

**Figure 2:**
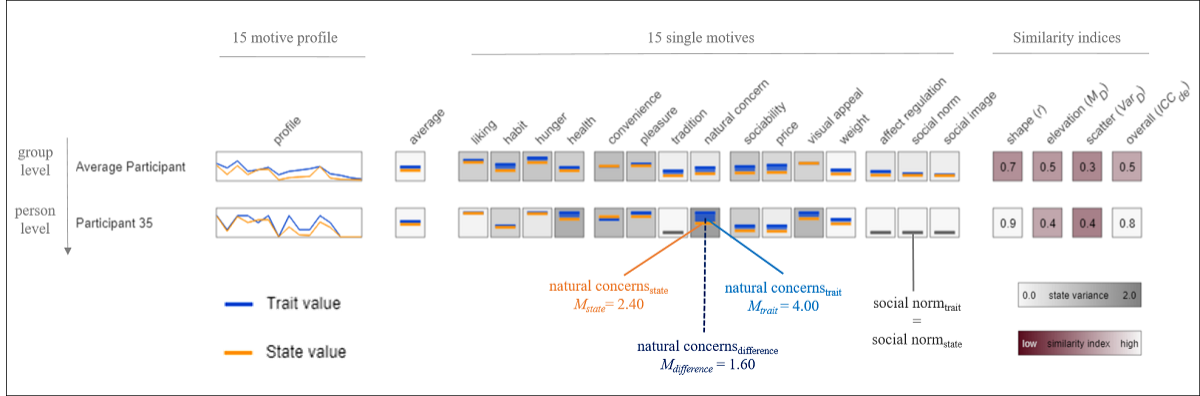
Illustration of the visualized person x motive data matrix and similarity indices for the 15 trait and state eating motive profiles. The first line displays data for the average participant (between-person level). The second line displays data for a single participant (No. 35) (within-person level). D=Difference score (trait value—state value).

The data from each participant and eating motive were visualized in a matrix using the SMART-Profile-Explorer, which is illustrated in Figure 2. Statistical indices and individual trait and state motive profiles are additionally summarized in Multimedia Appendices 2 to 4. The visualized person × motive data matrix encompasses 3 dimensions: (1) the between-person level, displaying aggregated state and trait motives across participants and motives, (2) the between-motive level, for comparing pairs of trait and state motives within each of the 15 motives across all participants (vertical comparison), and (3) the within-person level, allowing a comparison of the 15 trait and state eating motives within a single participant (horizontal comparison). The 3 profile similarity indices were calculated, respectively, and averaged means of the 15 trait and state eating motives are additionally displayed as the last lines of the matrix.

Within the visualized person × motive data matrix, for the group and for each participant, the first left column shows the individual trait (blue line) and state eating motive profile (orange line). The second column depicts the average trait (blue dash) and state (orange dash) values aggregated across the 15 eating motives for the group and each participant, respectively. Columns 3 to 17 display the 15 trait (blue dash) and 15 state (orange dash) eating motives separately. Values within each data box can range from 1 (dash at the bottom) indicating a low value for the respective motive to 4 (dash at the ceiling) indicating a high value for the motive. The difference between the trait and state motive values is further visualized by the size of a colored square between the trait and state motives. The greater the difference, the larger the colored square, whereas the depth of the square’s color is determined by how pronounced the motive is. In addition, the white-gray background of the motive data boxes changes to visualize the observed variability of each state eating motive. A darker shading indicates a greater variance across the longitudinal in-the-moment assessment for the state motive and, hence, a greater within-person motive fluctuation across time. In addition, columns 18 to 21 display the 4 similarity indices shape (*r*), elevation (*M_D_*), scatter (*Var_D_*), and the overall similarity index (*ICC_de_*), with lighter colors indicating a high similarity value and darker colors indicating a low similarity value on the respective index.

Illustrating the visualized data matrix exemplarily (see Figure 2) shows that participant 35 scored high on the overall similarity with *ICC_de_*=0.8. More specifically, the separate indices revealed *r*=0.9 for shape similarity as well as *M_D_*=0.4 for elevation and *Var_D_*=0.4 for scatter similarity. Focusing on the single eating motive *natural concerns,* participant 35 scored higher in the trait (*M*_trait_=4.00, blue dash) than in the state assessment (*M*_state_=2.40, orange dash). This difference of 1.60 is further visualized by the colored square. As participant 35’s trait motive for *natural concerns* was more pronounced than the state motive, the square is colored in blue. The comparatively dark gray background color of the data box indicates a high observed state variance across the in-the-moment assessment of *natural concerns* with *Var*=1.49 (for more details, see Multimedia Appendix 4).

### State and Trait Eating Motives: The Between-Person Level

**Figure 3:**
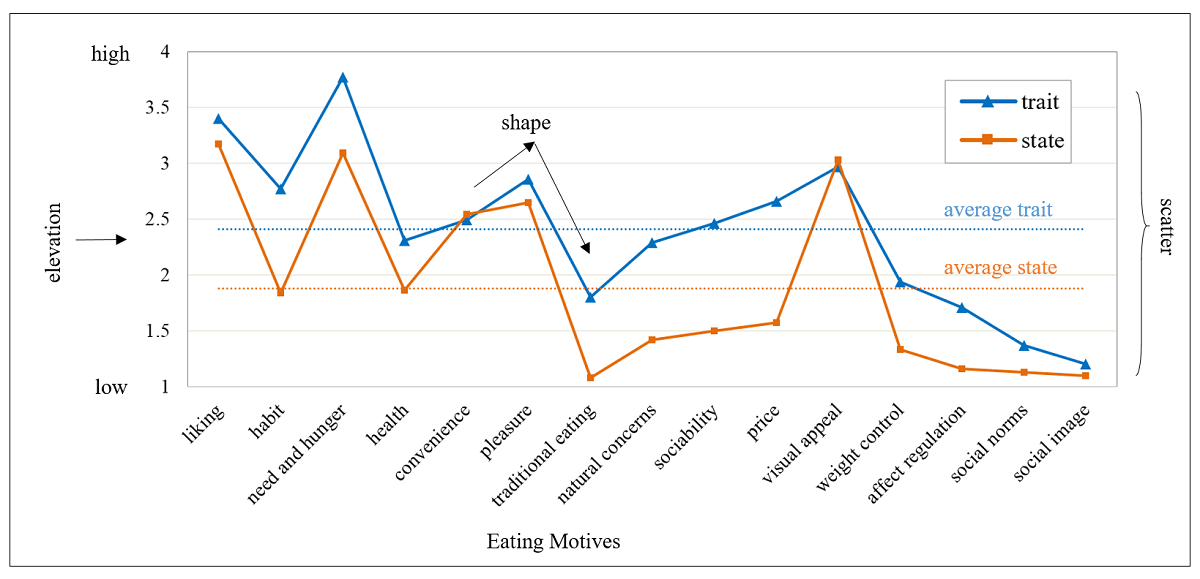
Average (typical) profile of the 15 trait and state eating motives with *ICC_de_*=0.52, *P*<001. Ratings ranged from (1) “strongly disagree” to (4) “strongly agree.” Motives are arranged according to their rank order observed for The Eating Motivation Survey.

Figure 3 illustrates the averaged eating motive profiles of the 2 assessment approaches. The omnibus index of profile similarity yielded a good overall similarity between the trait and state eating motive profiles across participants with *ICC_de_*=0.52 (*P*<.001). Thus, 27% of the observed variance in state eating motive profiles is explained by respective trait eating motive profiles.

The shape of the averaged trait and state motive profiles coincides with *r*=0.65, *P*<.001, indicating a comparable rank order across participants. However, trait and state motive profiles differed substantially in respect to the observed elevation (*M_D_*=0.53). Trait motives were rated higher on average than state motives, *M*_trait_*=*2.41, SD 0.31; *M*_state_=1.88, SD 0.23; *t*_34_=9.02; *P*<.001; *d*=1.97. In terms of scatter similarity, the average ratings ranged from 1.20 to 3.77 for trait and from 1.09 to 3.12 for state motives, indicating comparable scatters for both assessment methods. The average scatter index yielded a raw variance difference of *Var_D_*=0.33.

### State and Trait Eating Motives: The Between-Motive Level

Of 15 eating motives, 12 were rated significantly higher on average when assessed as trait motives as compared with state motives, indicating a consistent pattern of results with the found overall elevation differences. Large differences between trait and state eating motives (*d*>0.8) were found for *price*, *sociability*, *need and hunger*, *traditional eating*, *habit*, and *natural concerns*. Moderate mean level differences (*d*>0.5) emerged for *weight control*, *affect regulation*, *health*, *pleasure*, and *liking,* whereas a small effect size (*d*>0.3) emerged for *social norms*. Despite these elevation differences, positive correlations were found for 9 out of 15 eating motives, indicating that participants who had a higher trait eating motive also tended to exhibit higher average state scores for the respective eating motive (for more details, see Multimedia Appendix 2). The highest correlations between state and trait eating motives (*r*≥0.50) were found for *visual appeal*, *weight control*, *affect regulation*, *natural concerns*, and *price*. State-trait correlations within the medium effect size range (*r*>0.30) were observed for *pleasure*, *sociability*, *convenience*, and *health*. Comparing the variability for each pair of trait and state eating motives showed that some motives such as *sociability*, *weight control*, *traditional eating*, and *affect regulation* showed large variances in the trait (*Var*≥0.64) but low variances (*Var*≤0.19) in the state assessment. Inversely, motives such as *visual appeal*, *pleasure*, *convenience*, *health*, and *natural concerns* showed large variances (*Var*≥0.67) in both the trait assessment and the state assessment (*Var*≥0.41), indicating relatively high situational and/or between-person fluctuations.

### State and Trait Eating Motives: The Within-Person Level

**Figure 4:**
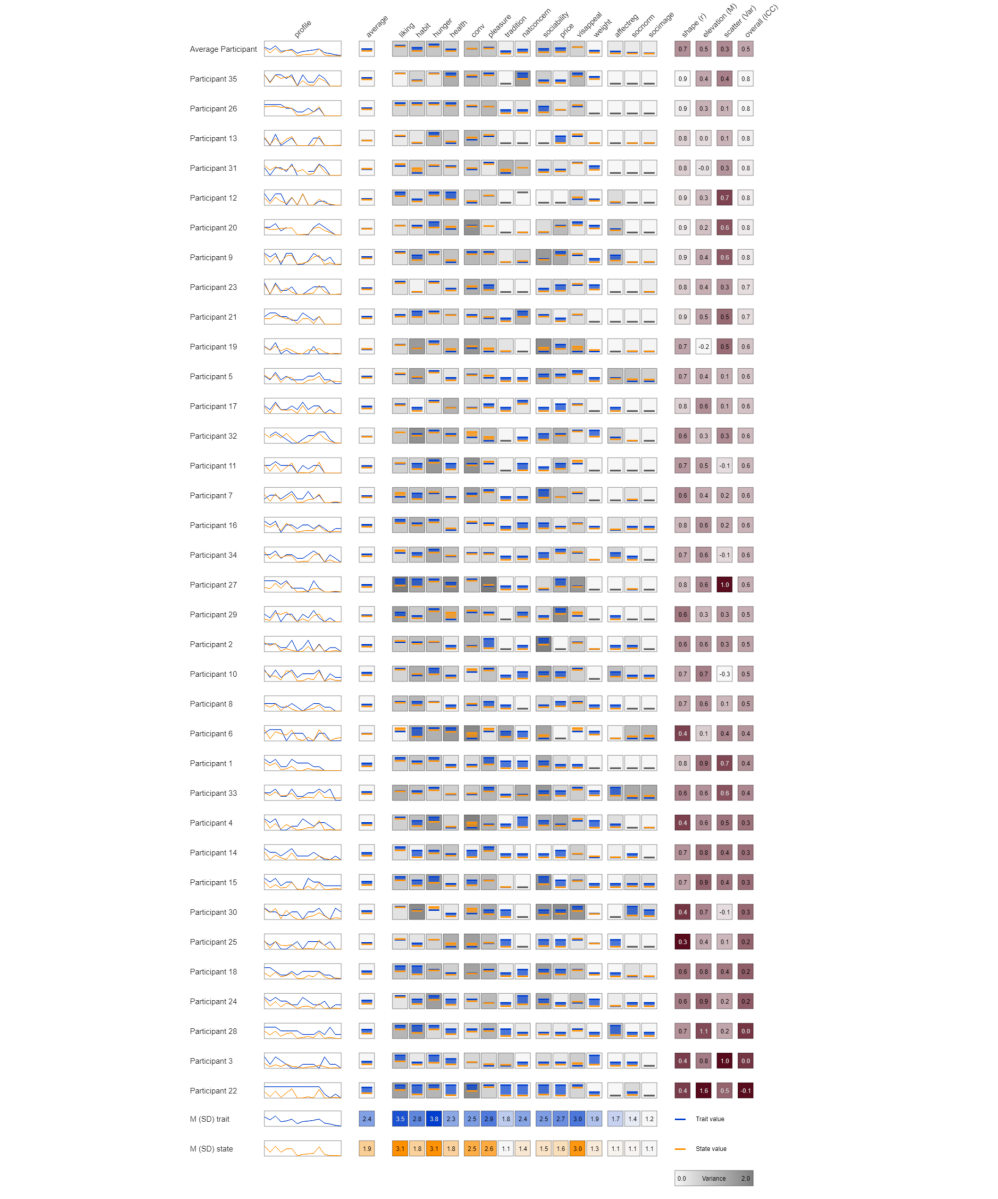
Visualized full person x motive data matrix. Participants are arranged in descending order according to the overall motive profile similarity (*ICC_de_*) from high (top) to low (bottom).

In Figure 4, participants are arranged according to their overall profile similarity (*ICC_de_*), with participant 35 displaying the highest and participant 22 the lowest profile similarity. This omnibus index yielded a high overall similarity for 7 of the 35 participants, with *ICC_de_*≥0.80. Thus, at least 64% of the observed variance in the state profile was explained by the respective trait profile. In addition, 15 participants showed a substantial profile similarity with at least 25% of the observed state variance explained by the trait variance (*ICC_de_*≥0.50). However, the remaining 13 participants showed only a low overall similarity between state and trait eating motive profiles, with less than 25% of the variance explained. The shape of the individual trait and state motive profiles coincides with *r*≥0.80 for 13 participants, indicating a highly similar rank order of the 15 eating motives within these participants. Furthermore, 16 participants showed a substantial rank order similarity with *r*≥0.60, and 6 participants showed a comparable low shape similarity with *r*≤0.40. Trait and state eating motive profiles differed substantially within participants for the observed elevation. At both group and individual levels, trait motives were rated higher on average than state motives. Specifically, 20 of the 35 participants scored half a point higher on the 4-point rating scale in the trait compared with the state assessment, whereas only 2 participants rated the state motives (slightly) higher than the trait motives. Comparing the variability between state and trait eating motives by using the index scatter shows substantial interindividual differences in intraindividual trait-state similarity. A total of 11 participants showed an average overall raw variance difference of *Var_D_*≥0.5, indicating a substantial scatter difference between state and trait motives within these participants. Conversely, 13 participants showed a difference in variance of 0.2 or lower.

In addition, comparing the 4 similarity indices at the intraindividual level shows marked interindividual differences. For example, the eating motive profiles of participants 35 and 13 yielded the same overall similarity (*ICC_de_*=0.80), but their elevation and scatter values still differed. Comparing their motive profiles shows that the differences were located at different state-trait motive pairs. While participant 35 overestimated the importance of *natural concerns* when asked about the usual relevance for choosing food as compared with the relevance in the concrete eating situation, participant 13 showed an overestimation for the importance of *price* for his or her daily food choices. Hence, zooming in at the motive and person level revealed distinct individual similarity patterns leading to considerable differences in similarity between eating motives, as well as between and within individuals.

## Discussion

### Principal Findings

This study examined differences between why people think they eat what they eat (trait motives) and why they eat in the moment of consumption (state motives) by assessing 15 different basic eating motives measured by (1) a single timepoint dispositional (trait) and (2) an in-the-moment (state) assessment using smartphone-based EMA.

Examining the aggregated EMA data across eating occasions and participants, we found that in-the-moment assessed eating motives generally mirrored eating motives assessed through the classical single timepoint approach. Specifically, at the between-person level, the similarity indices including overall similarity, shape, and scatter indicated a comparable rank order between motives and a comparable variance pattern. A positive relationship was also found at the between-motive level, indicating that individuals who scored higher in the dispositional assessment also scored higher when assessing the same motive in the moment of eating.

However, the single timepoint assessed trait motives clearly differed in their mean level from in-the-moment assessed state eating motives, as indicated by the similarity index elevation. Compared with the state assessment, the trait assessment significantly overestimated 12 out of 15 eating motives. Interestingly, not only core motives such as *need and hunger*, *price*, *habit*, *sociability* but also motives such as *natural concerns* or *traditional eating* were rated far more importantly when people indicated why they typical eat than when asked in the moment of consumption. Similarly, motives such as *health*, *weight concerns*, or *affect regulation* were overestimated in the trait assessment. Mean levels for state and trait motives only concurred for the motives *convenience*, *visual appeal*, and *social image.* *Convenience* and *visual appeal* were both important reasons for eating, and the high mean values in the state and trait assessment indicate that they are generic motives, influencing eating on most occasions, across participants, and in a wide range of different contexts. Conversely, the low observed mean values for *social image concerns* in both assessments suggest that these concerns are limited to specific eating situations. This is in line with research on impression management, which suggests that eating behavior can serve a role of showing oneself to be a particular type of person in certain social situations [74-76]. For example, in specific situations such as eating with an unfamiliar man, women are more inclined to create an impression of femininity by restricting food intake to increase the desirability by presenting a feminine social identity [74].

Analyses on the within-person level showed pronounced interindividual differences in intraindividual patterns between trait and state motives (see also Figure 4). Some participants had a very good notion of why they eat, as their trait and state motives converged. For example, participant 35 gave highly accurate estimations for 14 out of 15 motives. Only sustainability concerns were less characteristic in the moment of eating as participant 35 believed when gauging his/her typically eating motives. However, other participants (eg, participant 22) showed a considerable divergence across almost all 15 eating motives. Thus, using an in-the-moment assessment of eating motives suggests that people show clear discrepancies between why they think they eat and why they actually eat in-the-moment.

These discrepancies between experienced and remembered eating motives are of crucial importance for future interventions. Research indicates that what we remember seems to be more predictive for our future behavior than what we experience [45,46,77-81]. However, as this research shows, remembered (ie, dispositional) eating motives do not accurately represent in-the-moment experiences. For instance, one might assume that health concerns primarily guide one’s own eating behavior, but when it comes to the actual moment of eating, taste or visual appeal of a tempting food affects the actual food choice to a higher degree. Identifying and addressing these discrepancies between why people think they eat and why they actually eat in the moment indicates important starting points for changing eating behaviors. Especially, when we aim to implement mHealth interventions that are characterized by acting *in-the-moment*, it is of crucial importance to target processes and experiences of the eating situation itself.

### Implications of These Findings

The observed overestimation of the influence of motives in the moment of consumption through dispositional assessments might reflect different methodological issues affecting single timepoint measurement of trait motives and longitudinal assessment of state motives, heterogeneous mechanisms and causes, or the complexity and multidimensionality of eating behavior in day-to-day life [82,83]. One might argue that people tend to view themselves favorably and therefore, overestimate the typical eating motives that they see as desirable. A recent study showed that the 15 eating motives differ in their perceived desirability [49], with *hunger*, *health*, and *liking* being perceived as highly desirable motives, whereas *social image*, *social norms*, and *affect* *regulation* are seen as particularly undesirable. For desirable motives, participants rated their own motives as higher than their peers, whereas the opposite pattern emerged for undesirable motives, indicating unrealistic optimism in eating motives [49]. In this study, trait eating motives were generally more pronounced than state motives, including desirable (eg, need and hunger) and undesirable ones (eg, affect regulation). Hence, social desirability concerns are unlikely to explain the observed pattern of results. Admittedly, to derive a judgment about their typical eating motives, people need to recall and aggregate the different reasons across multiple eating occasions. Numerous studies have shown that people often use heuristics to form judgments about their behavior and characteristics [84-86]. In particular, participants might have used the representativeness heuristic as a mental shortcut to evaluate their typical eating motives, which might have caused the observed overestimations. In addition, measurement issues may have inflated the magnitude of trait motives or deflated the magnitude of state motives. For example, response biases to the mobile EMA scales such as underreporting of momentary experiences of eating motives across assessments might have contributed to the lower mean levels. Furthermore, enduring trait motives could shape the actual eating behavior and as a consequence limit the occurrence of specific state eating motives. For example, an individual with a pronounced weight control motive at the trait level might avoid tempting food choices altogether (eg, sweets and snacks) and therefore, she or he is less likely to report state weight control motives. Although avoidance of food items or eating situations might be caused by motives such as weight control, tradition, or social norms, in most cases, people probably opt for alternatives (eg, an apple instead of chocolate) and thus, trait and state motives would covary.

To consider the real-life situational fluctuations in eating behaviors [15,33,87] and prevent retrospective recall biases [45,77,88], a smartphone-based EMA approach was used to assess eating motives in the moment. Although it is admittedly true that in-the-moment approaches offer advantages over conventional single timepoint methods, especially in terms of their ecological validity [54,89], they are also accompanied by increased expenditure for both participants and researchers. Especially in the case of eating, participants must log every eating occasion over a prolonged period to generate representative data, which in turn leads to methodological and statistical challenges for researchers [90]. For instance, research is challenged with finding new, elaborated methods of analyzing the resulting high-dimensional data [90,91]. Developing methods that force data analyses to go beyond aggregated mean values and consider the between- and within-person levels is, therefore, an important achievement in the field of mHealth.

The additional analysis on the person and motive level, which was facilitated through the SMART-Profile-Explorer, acknowledges these person- and situation-specific differences in eating motives. The interplay between inter- and intraindividual differences that emerged from these findings could only result from a comprehensive analysis that incorporates different aggregation levels, rather than focusing on overall means and between-person effects. The implemented visualized person × motive data matrix fosters this approach by facilitating an analysis that is close to the raw data. This approach aims to not only increase data transparency in terms of open data appeals [68] but also to illustrate underlying patterns and dynamics of eating motives. Focusing not just on between-person but also on within-person variability is crucial for gaining more detailed insights into psychological processes and counteracting the “threat to the conceptual integrity” of psychological research elicited through a mismatch between theory and research practice [92]. We are convinced that a deeper understanding of human food choice behavior can only be achieved by integrating between- and within-person effects and combining findings at the motive and person level.

### Strengths and Limitations

To our knowledge, this study is the first that directly compares a single timepoint dispositional assessment of eating motives with an in-the-moment assessment of the same motives in the same individuals by using a smartphone-based EMA to derive conceptual conclusions. Furthermore, these findings are indicative for planning and designing effective health interventions. To promote healthy eating behavior and counteract the associated health risks of the rising obesity epidemic [93-95], the interplay between person, situation, and eating motive needs to be considered to improve intervention effectiveness by identifying critical cues, moments, and target groups.

Although these findings expand the current state of research and provide important implications for health interventions, they must be viewed in consideration of 2 main limitations that should be accounted for in future research. Although the sample size is comparable with other studies in eating research [15,22,96,97], it is small, and the participants were predominantly white, female, and highly educated. Moreover, future research is needed to investigate preceding situations that might also determine eating behavior, such as buying, choosing, or preparing food, to draw reliable conclusions and shed further light on explanations for the differences found between dispositional and in-the-moment eating motives.

### Conclusions

In general, this study found a substantial overlap between the dispositional and in-the-moment assessment regarding eating motives. However, elevation markedly differ between the 2 assessment approaches and the majority of eating motives are overestimated in the dispositional assessment. A more detailed analysis of the interplay between person and motive revealed interindividual differences in intraindividual similarity patterns. Hence, for a comprehensive understanding of why we eat what we eat, dispositional assessments need not only to be extended by comprehensive EMAs that take place in the moment but also to be analyzed at the between- and within-person level. Capturing these individual dynamics in eating motives is crucial to develop tailored dietary interventions to intervene in the critical moments of situations that determine eating behavior.

## Appendix

Multimedia Appendix 1 Single-item version of The Eating Motivation Survey.

Multimedia Appendix 2 Statistical characteristics of trait and state eating motives on the between-motive level.

Multimedia Appendix 3 Profiles of the 15 eating motives for each individual.

Multimedia Appendix 4 Profile similarity indices for trait and state eating motives at the within-person level.

## Abbreviations

EMA: ecological momentary assessment
FCQ: Food Choice Questionnaire
ICC_de_: double-entry intraclass correlation
mHealth: mobile health
TEMS: The Eating Motivation Survey
