# Supplementary material for: Why We Eat What We Eat: Assessing Dispositional and In-the-Moment Eating Motives by Using Ecological Momentary Assessment
Source: JMIR Mhealth Uhealth. 2020 Jan 7;8(1):e13191. doi: 10.2196/13191 (PMC6996745; doi:10.2196/13191)
Supplement: Multimedia Appendix 1 [file mhealth_v8i1e13191_app1.pdf]

Multimedia Appendix 1

Single-item version of The Eating Motivation Survey (TEMS; Renner et al., 2012).

Ernährungsmotiv (Original version)<br>*Eating motive (Translated version)*	‚Warum haben Sie das gegessen?‘ (State)<br>*‘I eat what I eat…’ (State)*<br>‚Warum essen Sie das was Sie essen?‘ (Trait)<br>*‘I eat what I eat…’ (Trait)*	‚trifft nicht zu‘<br>*‚strongly disagree‘*			‚trifft voll zu‘<br>*‚strongly agree‘*
**Appetit**<br>*Liking*	…Appetit<br>*...because I like it*	◯	◯	◯	◯
**Gewohnheit**<br>*Habits*	…aus Gewohnheit<br>*...because I usually eat it*	◯	◯	◯	◯
**Hunger**<br>*Need and Hunger*	…Hunger<br>*...because I´m hungry*	◯	◯	◯	◯
**Gesundheit**<br>*Health*	…aus gesundheitlichen Gründen<br>*...because it’s healthy*	◯	◯	◯	◯
**Einfachheit**<br>*Convenience*	…geringer Aufwand<br>*...because it’s convenient*	◯	◯	◯	◯
**Genuss**<br>*Pleasure*	…um mir eine Freude zu machen<br>*...to indulge myself*	◯	◯	◯	◯
**Tradition**<br>*Traditional Eating*	…aus traditionellen Gründen (z.B. Fest)<br>*...because it’s a tradition (e.g. a special occasion)*	◯	◯	◯	◯
**Natürlichkeit**<br>*Natural Concerns*	…aus ethischen Gründen (z.B. fairer Handel)<br>*...for ethical reasons (e.g. fair trade)*	◯	◯	◯	◯
**Gemeinschaft**<br>*Sociability*	…weil es gesellig ist<br>*...to be sociable*	◯	◯	◯	◯
**Preis**<br>*Price*	…aus preislichen Gründen<br>*...because it is inexpensive*	◯	◯	◯	◯
**Präsentation**<br>*Visual Appeal*	…weil es mich angesprochen hat<br>*...because it spontaneously appeals to me*	◯	◯	◯	◯
**Gewichtskontrolle**<br>*Weight Control*	…zur Gewichtskontrolle<br>*...because I watch my weight*	◯	◯	◯	◯
**Affektregulation**<br>*Affect Regulation*	…aufgrund von negativen Emotionen (z.B. Frust)<br>*...to help me feel better (e.g. when I’m frustrated)*	◯	◯	◯	◯
**Soziale Normen**<br>*Social Norms*	…weil es von mir erwartet wurde<br>*...because others expected me to eat it*	◯	◯	◯	◯
**Soziales Image**<br>*Social Image*	…weil andere das gut finden<br>*...because others like it*	◯	◯	◯	◯
