# Supplementary material for: Why We Eat What We Eat: Assessing Dispositional and In-the-Moment Eating Motives by Using Ecological Momentary Assessment
Source: JMIR Mhealth Uhealth. 2020 Jan 7;8(1):e13191. doi: 10.2196/13191 (PMC6996745; doi:10.2196/13191)
Supplement: Multimedia Appendix 2 [file mhealth_v8i1e13191_app2.pdf]

Multimedia Appendix 2

Statistical characteristics of trait and state eating motives at the between-motive level.

	Mean								
	trait	*state*			*Paired t-test*				
	M(SD)	*M(SD)*	M_D *(SD)*	95% CI	*t*	*df*	*p*	*Cohen´s d*	*r*
Liking	3.50 (0.56)	3.12 (0.52)	0.39 (0.74)	0.13 - 0.65	3.08	33	.004	0.34	.079 ns
Habit	2.77 (0.88)	1.84 (0.58)	0.93 (0.90)	0.62 - 1.24	6.11	34	.000	1.23	.292 ns
Need and hunger	3.77 (0.43)	3.08 (0.46)	0.69 (0.62)	0.48 - 0.90	6.61	34	.000	1.35	.027ns
Health	2.31 (0.96)	1.83 (0.64)	0.48 (0.95)	0.16 - 0.81	3.01	34	.005	0.58	.354*
Convenience	2.49 (0.82)	2.54 (0.69)	-0.05 (0.85)	-0.34 - 0.24	-0.36	34	.724	-0.07	.376*
Pleasure	2.94 (0.92)	2.58 (0.71)	0.36 (0.84)	0.07 - 0.66	2.53	33	.016	0.44	.493**
Traditional Eating	1.80 (0.80)	1.08 (0.19)	0.72 (0.83)	0.43 - 1.01	5.09	34	.000	1.28	-.102 ns
Natural concerns	2.35 (1.10)	1.38 (0.69)	1.00 (0.90)	0.69 - 1.32	6.50	33	.000	1.03	.575***
Sociability	2.46 (0.92)	1.49 (0.39)	0.97 (0.84)	0.68 - 1.25	6.81	34	.000	1.25	.408*
Price	2.66 (0.84)	1.58 (0.59)	1.08 (0.74)	0.83 - 1.33	8.69	34	.000	1.45	.515**
Visual appeal	2.97 (0.82)	2.97 (0.64)	-0.00 (0.65)	-0.22 - 0.22	-0.02	34	.981	-0.00	.635***
Weight control	1.94 (0.94)	1.33 (0.44)	0.61 (0.74)	0.35 - 0.87	4.85	34	.000	0.70	.631***
Affect regulation	1.71 (0.89)	1.15 (0.24)	0.56 (0.77)	0.30 - 0.83	4.31	34	.000	0.65	.606***
Social norms	1.37 (0.65)	1.13 (0.18)	0.25 (0.63)	0.03 - 0.46	2.30	34	.028	0.48	.226 ns
Social image	1.20 (0.47)	1.09 (0.19)	0.11 (0.48)	-0.06 - 0.28	1.35	34	.187	0.30	.147 ns

Note. Rating of eating motives ranged from 1 'strongly disagree' to 4 'strongly agree'. *** $p < .001$, ** $p < .01$, * $p < .05$
