# Supplementary material for: Why We Eat What We Eat: Assessing Dispositional and In-the-Moment Eating Motives by Using Ecological Momentary Assessment
Source: JMIR Mhealth Uhealth. 2020 Jan 7;8(1):e13191. doi: 10.2196/13191 (PMC6996745; doi:10.2196/13191)

Multimedia Appendix 3

Profiles of the 15 eating motives for each individual with blue lines representing trait motives and orange lines state motives.

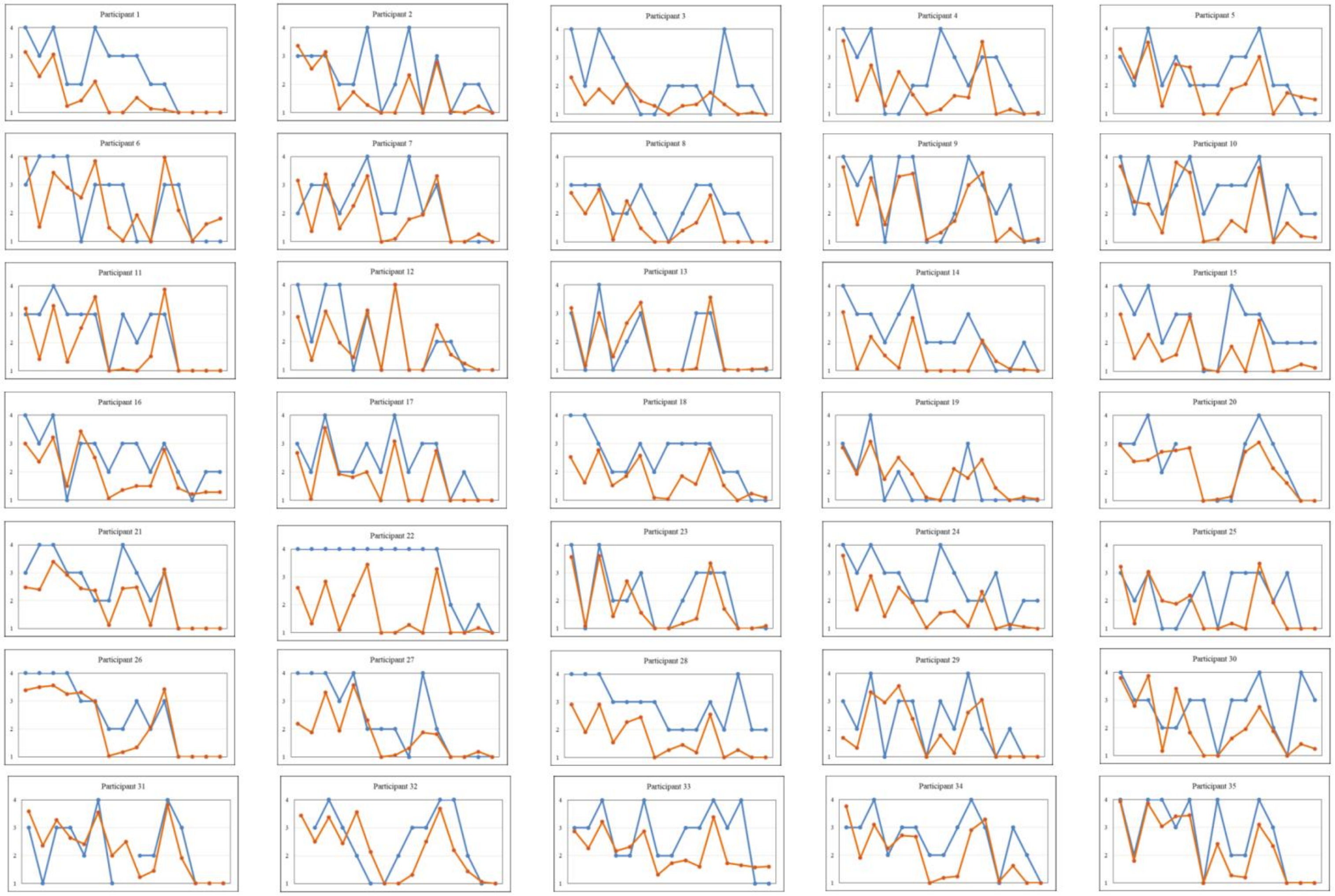

Supplement: Multimedia Appendix 3 [file mhealth_v8i1e13191_app3.pdf]
