# Supplementary material for: Why We Eat What We Eat: Assessing Dispositional and In-the-Moment Eating Motives by Using Ecological Momentary Assessment
Source: JMIR Mhealth Uhealth. 2020 Jan 7;8(1):e13191. doi: 10.2196/13191 (PMC6996745; doi:10.2196/13191)
Supplement: Multimedia Appendix 4 [file mhealth_v8i1e13191_app4.pdf]

Multimedia Appendix 4

Profile similarity indices for trait and state eating motives at the within-person level.

	Mean		*Var*		Shape similarity	Elevation similarity	Scatter similarity	Overall similarity
Participant	*trait*	*state*	*trait*	*state*	*r*	M_D	Var_D	ICC_{de}
35	2.67	2.25	1.67	1.29	.914***	0.42	0.38	.848***
26	2.53	2.20	1.41	1.31	.885***	0.34	0.10	.844***
13	1.80	1.77	1.17	1.07	.813***	0.03	0.10	.811***
31	2.21	2.25	1.26	0.98	.807***	-0.03	0.27	.804***
12	2.13	1.88	1.70	0.98	.857***	0.26	0.71	.802***
20	2.29	2.05	1.30	0.67	.884***	0.23	0.62	.797***
9	2.53	2.14	1.70	1.10	.863***	0.40	0.59	.789***
23	2.13	1.77	1.27	1.00	.800***	0.36	0.27	.741***
21	2.47	2.02	1.27	0.77	.854***	0.45	0.50	.736***
19	1.60	1.80	0.97	0.48	.702**	-0.20	0.49	.636***
5	2.40	2.03	0.83	0.71	.714**	0.37	0.12	.633***
17	2.33	1.72	0.95	0.81	.817***	0.61	0.14	.629***
32	2.43	2.18	1.34	1.01	.648*	0.25	0.33	.595**
11	2.33	1.85	1.10	1.22	.670**	0.48	-0.12	.584**
7	2.27	1.89	1.07	0.90	.633*	0.38	0.16	.571**
16	2.53	1.96	0.84	0.68	.754**	0.57	0.16	.568**
34	2.60	2.04	0.83	0.93	.710**	0.56	-0.1	.563**
27	2.40	1.76	1.69	0.68	.766**	0.64	1.00	.551**
29	2.20	1.92	1.17	0.92	.584*	0.28	0.25	.547**
2	2.27	1.70	1.07	0.76	.645**	0.56	0.30	.497**
8	2.20	1.62	0.60	0.52	.707**	0.58	0.08	.467**
10	2.80	2.06	0.89	1.14	.716**	0.74	-0.26	.496**
6	2.40	2.28	1.54	1.18	.446	0.12	0.36	.438*
1	2.40	1.53	1.26	0.57	.778**	0.87	0.69	.409*
33	2.73	2.15	1.07	0.43	.609*	0.59	0.63	.383*
4	2.40	1.76	1.26	0.80	.443	0.64	0.45	.293
14	2.33	1.49	0.95	0.51	.654**	0.84	0.44	.287
15	2.60	1.65	0.97	0.55	.696**	0.95	0.42	.268
30	2.73	2.05	0.92	1.05	.414	0.68	-0.12	.254
25	2.13	1.73	0.84	0.76	.307	0.4	0.08	.239
18	2.53	1.74	0.84	0.41	.566*	0.79	0.43	.206
24	2.67	1.72	0.81	0.62	.598*	0.94	0.19	.195
28	2.87	1.72	0.70	0.52	.657**	1.15	0.18	.041
3	2.20	1.44	1.17	0.16	.436	0.76	1.01	.039
22	3.33	1.69	1.38	0.85	.434	1.64	0.53	-.136
total	2.41	1.88	1.14	0.81	.648. ***	0.53	-0.33	.524***

Note. Similarity refers to the comparison of trait and state assessed eating motives.

*** *p<.001, **p<.01, *p<.05*
